# Nano–bio surface interactions, cellular internalisation in cancer cells and e‐data portals of nanomaterials: A review

**DOI:** 10.1049/nbt2.12040

**Published:** 2021-03-22

**Authors:** Ram Dhan Yadav, Abha Chaudhary

**Affiliations:** ^1^ Department of Biochemistry Panjab University Chandigarh India; ^2^ Department of Chemistry Government Post Graduate College Ambala Cantt Haryana India

## Abstract

Nanomaterials (NMs) have abundant applications in areas such as electronics, energy, environment industries, biosensors, nano devices, theranostic platforms, etc. Nanoparticles can increase the solubility and stability of drug‐loaded materials, enhance their internalisation, protect them from initial destruction in the biological system, and lengthen their circulation time. The biological interaction of proteins present in the body fluid with NMs can change the activity and natural surface properties of NMs. The size and charge of NMs, properties of the coated and uncoated NMs, nature of proteins, cellular interactions direct their internalisation pathway in the cellular system. Thus, the present review emphasises the impact of coated, uncoated NMs, size and charge, nature of proteins on nano–bio surface interactions and on internalisation with specific focus on cancer cells. The increased activity of NPs may also result in toxicity on health and environment, thus emphasis should be given to assess the toxicity of NMs in the medical field. The e‐data sharing portals of NMs have also been discussed in this review that will be helpful in providing the information about the chemical, physical, biological properties and toxicity of NMs.

## 1 INTRODUCTION

In the field of nanomaterials (NMs), nanoparticle interaction with the natural frameworks has become a quickly developing powerful field of exploration. Understanding this interaction will help to comprehend the effect and toxicity of NMs on human health and environment. A nanoparticle is characterised as a material having a measurement of 1–100 nm diameter. The nanoparticles (NPs) are formed due to the congregation of gathering particles or atoms. As NPs show novel physical and chemical properties, these are very unique in relation to those of bulk material and have accordingly increasing and expanding significance in different fields like agribusiness, aviation, material, energy and biomedical fields, including drug delivery, device‐based therapy, tissue engineering and medical imaging. The interaction of NMs with the biological systems, that is, nano–bio interactions is being developed into a fast emerging field of research. As the targeted NPs enter into the body, nano–bio interactions take place at every scale (e.g. interactions with proteins, DNA, macrophages or blood vessels) or during their in vivo transport. Therefore, complete understanding of nano–bio interactions is essential to make a progress in targeting efficiency of NMs, for detection, therapeutic efficiency as well as for the approval of nanomedicines [1].

There are many interactions that appear between the NPs surface and biological system. These are molecular, chemical, electrical, mechanical interactions that result into the exchange of information between surface of NPs and biological components (proteins, membrane phospholipids, endocytic vesicles, organelles such as mitochondria, nucleus and biological fluids) [2]. The internalisation of NPs through the bio‐membrane is carried out by their interaction with different proteins [3]. In some cases, these nano–bio interactions are not favourable and do not allow the NPs to cross the lipid bilayer. Knowing the interaction of NPs with the cells as an entryway into the body is crucial for nanotoxicology and drug delivery as this interaction directs the systemic absorption of NMs and their toxicity towards specific organs. NMs interaction and toxicity are decided during their synthesis or during the functionalisation of NMs. Attachment or adsorption of natural or synthetic molecules or proteins on NMs can produce different types of interactions, toxicity, and can change the fate of NPs during in vivo study.

Many interactive forces drive the ideal biotic and abiotic interface interactions of biological systems with NPs, for example, Brownian forces, hydrodynamic and electrostatic forces. Hydrodynamic interactive forces decrease the fluid velocity relative to the solid object. These forces increase with proximity and increase the frequency of collisions between nano–bio surface and enhance the interactions as these are able to overcome electrostatic repulsion, thus push the NPs closer to the cell membrane [4]. Due to the electrostatic interactions, the NPs gain an electrical double layer in solution. These coulombic forces operating at the surface are although weak, but they strongly influence the biological process [5]. Van der Waals forces operate between the particle surface due to solvent interactions also. These forces either enhance the uptake or resist the acceptance of NPs depending upon the biophysical interactions. Therefore, the nature of designing and functionalisation of NPs’ surface should be such that it minimises the resistive forces and increases the uptake of NPs.

The distinctive properties of NPs such as smaller size and high surface area show their significant potential and utilization in biomedicine. The change of the size, charge and surface arrangement of NPs can direct their internalisation pathway, aiding the NPs to escape lysosomes and help them to interact directly with the target area [6]. Similar nano–bio surface charges repel and dissimilar charges attract each other. The positive charge induces fast membrane depolarisation across different cell types and thus rapid uptake occurs inside the cell. Soluble NPs are stable in the solvent, prevents aggregation, and form sterically stabilised transient NPs dimer. The lyophilic surface of the NPs enhances solubility and safeguards them against plasma protein binding during their delivery. The lyophobic surface is more active and energetic against the biological fluid and membrane [7]. Moreover, a comprehensive knowledge of how physicochemical parameters of NPs impact their interaction with the biological systems is necessary for their safe and effective applications in medicine [8]. For the past few years, NPs have been conjugated with real‐time imaging agents like dyes, quantum dot, fluorescent materials etc. to target the cancer receptors. These types of nano‐modification determinations could not have truly been fruitful without understanding the interaction of NPs inside the cellular system. The diffusion of NPs into lipid bilayers and cell membranes has significant effects on controlled drug release and thus on medical cures [9]. The decorated NPs may act as carriers for targeted drug delivery, stimulating the controlled release and effective absorption of drug with increase in the therapeutic effects and decrease in the adverse effects.

Gehr et al. gave brief idea about how entry of NPs into organ tissues get affected by internal blood–tissue barriers [10]. Rahim et al. discussed about the mechanisms of interaction of only green NPs with cells, with different organs and organelles [11]. Cheng et al. explained about metallic NPs but the interaction of NPs with protein corona was not very well explained [12]. Behzadi et al. well elaborated different mechanisms of endocytosis for NPs’ entry inside the cell, for example, phagocytosis, clathrin‐mediated endocytosis, caveolin‐mediated endocytosis, clathrin/caveolae‐independent endocytosis and macropinocytosis. In addition to that, some non‐endocytic entry mechanisms were also concisely summarised [13]. They also explained about the techniques which can be utilised to study cellular internalisation and trafficking of NPs.

There are chances of generation of a number of nano–bio interactions when NMs with different physical and chemical properties (size, shape, charge, surface area, accumulation, crystallinity and decoration of surface) communicate with several biological moieties (like proteins, lipids, cells, tissues cell membranes, organelles and organs), it is very difficult to explain each part of nano–bio interactions. In continuation of our earlier works, on the synthesis and functionalisation of NMs (to check their anticancer potential against human lung carcinoma (A549) and ovarian cancer (CHO‐K1) and SiHa cell lines [14, 15, 16], in this review, we specifically discussed how the size, charge, coating or functionalisation of NPs (in the form of polymer, proteins or different functional groups) affect the interactions of NPs with the biological surface and also impact the cellular uptake and interaction of targeted and non‐targeted NPs with more emphasis on cancer cells. Moreover, it is important that the large amount of data for the properties, synthesis and study of toxicity of NPs should be available. Bai et al. in 2017 provided the names of e‐databases of NMs [17]. In this review, we have given information about e‐data portals, where the data about the preparation, properties and toxicity of NMs are available along with the purpose of these portals.

## 2 EFFECT OF SIZE AND CHARGE ON THE NANO–BIO SURFACE INTERACTIONS

**FIGURE 1:**
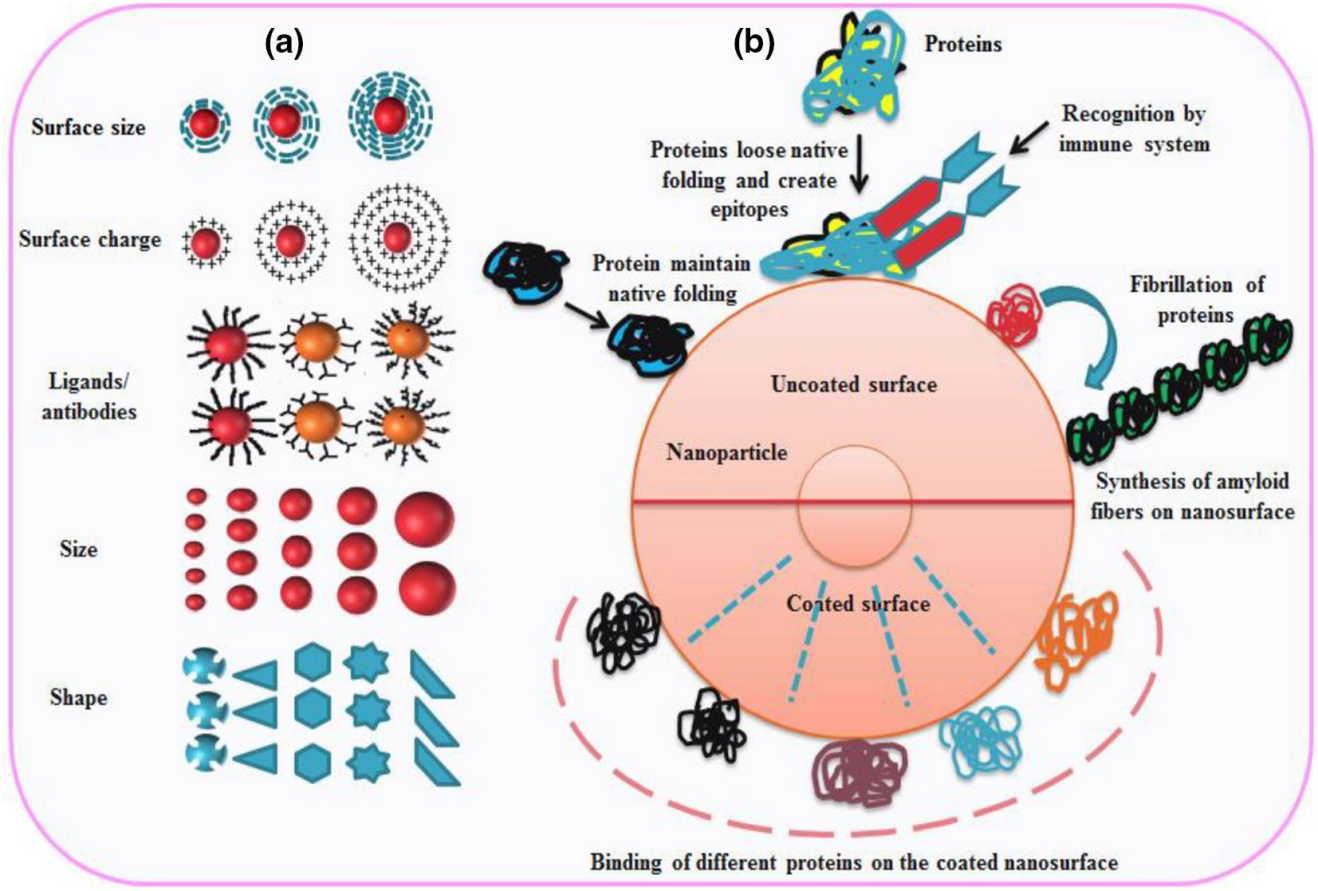
Diagrammatic images of physical properties of nanomaterials (a): Several properties like surface charge, shape, size and surface modification of nanomaterials directly or indirectly link with the efficacy and the toxicity of the NMs; the nature of protein and conformation changes after coming in contact with the nanosurface (b): The formation of protein corona, stabilisation and destabilisation depends upon the nanosurface and forces functioning at the time of interaction. The coated nanosurface with soluble materials may not always avoid the interaction with protein; though the uncoated nanosurface induces fibrillation of protein and produces epitopes recognised by the immune system

In a cellular system several factors like ion concentration, pH, active proteins, and surface charge of plasma membrane and size and charge of NPs are the crucial determinants that affect the interactions of NPs with the biological surface (Figure 1). The smaller size of NPs ensures numerous surface properties closer to the interface and a high surface‐active energy, which could differentiate them from mass materials. Due to high surface energy, the NPs have the ability to conjugate with the surface particles and to combine with other atoms leading to adsorption of proteins and high nano‐catalysis reaction chemistry and biological interactions. The high free energy on the surface of NPs can bring conformational and structural changes in the important organs leading to injury and damage to them.

With smaller particle size, the surface area of NPs basically expands and their atomic coordination number is decreased. As the particle size decreases, the volume increases, and a larger proportion of atoms appear at the nanosurface in comparison to those which appear inside the core [18]. NPs having 30 nm size can hold 5% of its atoms on its nanosurface, while that of 10  and 3 nm sized can hold 20% and half of their atoms, respectively. The decrease in the particle size to the nano‐size deliberately changes the physical properties (such as optical, thermal, magnetic, and mechanical properties) of NPs. As the size of NPs reduces to <10 nm, the melting point particularly increases due to the modification in chemical structures at nanoscale level [19]. Smaller sized particles show high toughness and malleability due to the strong Van der Waals forces [6].

The inherent property of changeability of size of gold nanoparticles (GNPs) aids their faster absorption and allows them to pass across the body circulatory system easily. Carboxymethyl xanthan gum capped GNPs were synthesised by Alle et al. with a smaller range of size (8–10 nm); the loading of doxorubicin on these NPs showed higher interaction, cellular uptake and cell apoptosis properties [20]. The study on the transport and interaction of NPs in kidneys also showed the importance of the size of NPs. The endothelium (70–90 nm pore size) restricts the entry of particles with hydrodynamic diameter more than 100 nm, while the glomerular basement membrane blocks the particles with hydrodynamic diameter less than or equal to 100 nm [1]. The large‐sized NPs can fragment into smaller NPs in the blood or in kidney glomerulus that ease their passage from kidney. The multiple layers of the glomerular filtration membrane lets the passage of modified NPs of hydrodynamic diameter (HD) of 1–6 nm easily into urine depending upon their size. The clearance efficiency of cysteine‐coated quantum dots was described through kidney increases as the size decreases. The HD of 5.52–4.99 nm and 4.36 nm size cysteine‐coated quantum showed clearance efficiency of 43.65% injected dose (ID) to 62.18% ID and 75.13% ID, respectively, 4 h after intravenous injection [16]. Likewise, PEG‐coated silica NPs were used containing HDs of 3.3 nm that are excreted rapidly than the NPs of 6.0 nm HD [21].

NPs’ size was found to be a major factor for regulation of blood circulation which is related to the tumour growth, tumour retention, and discharge of the drug [22]. The liposomes more than 200 nm size did not enter into the tumours when these are assessed in size from 40  to 700 nm. This was further confirmed by using a PEGylated unilamellar liposome of 50–150 nm diameter range that showed the highest circulation time, thereby leading to an improvement in the accumulation of tumours leading to the antitumour activity. Notably, the dependence of in vivo nature of NPs on their size shows expected outcomes established on the basis of discrete properties of each tumour and on the different areas within the same tumour. Using 10 nm GNPs, Arvizo et al. suggested that the cellular membrane potential contributes significantly to the mode of uptake, and it proved that the cationic GNPs were much more efficiently taken up by cancer as well as by primary normal cells than GNPs with anionic or neutral ligands because positively charged particles depolarise the membrane more efficiently [23].

In the cancer cells, Abduljauwad et al. found that the extreme secretion of lactate ions and sialic acid result into the elimination of positive ions from the cell surface to the intracellular space, and then the cell surface display the negative charges [24]. It was determined that in comparison to the normal cells, the cancer cells have more non‐specific van der Waals and electrostatic forces and have a higher negative surface charges. The surface charge of cell membrane controls the intracellular pathways, intracellular Ca^2+^ ion concentration and cell cycle etc. The nature of the charge also drives the different internalisation pathways for the uptake and transportation of NPs. Peter et al. described that positively charged GNPs of 4.6 nm size enter into the HeLa cells more effectively than negatively charged particles [9]. The positively charged NPs are taken up and transported by the calveolae/clathrin pathway, however, the possibility of entry of NPs directly by nonregulated means via lipid bilayer of the plasma membrane was disqualified and the entry by the chance of damage of membrane was also found ineligible.

The passage of the NPs across the extracellular matrix is very complex due to the characteristic mesh‐like arrangement. The extracellular matrix discards the particles if their diameters are more than the dimensions of the matrix, though it lets the smaller particles to transport through it. The larger particles are rejected by collagen fibrils of the matrix with 20–40 nm space [25]. Similarly, the basement membrane of extracellular matrix (ECM) hampers the passage of NPs on the basis of their size. Actually, the specific complex of ECM proteins significantly influence the transmucosal transportation of NPs. The passage and cellular uptake of NPs are affected by the charge of NPs and also on the charge of the ECM network. Besides size, the charge of the NPs add significantly to the behavioural properties of NPs in the biological systems [26]. The entry of NPs is blocked by negatively charged chondroitin sulphate component of ECM. While, higher toxicity was shown by carbonate form coated double hydroxide NPs constituting positively charged brucite‐like coatings in comparison to the chloride form for causing oxidative stress, cell death and membrane damage [27]. Nanotechnologists have also tried to reduce the negative zeta potential by modifying it into positive for the rapid uptake of NPs and for controlling the toxicity [9].

It was reported that due to smaller size, the NPs have greater permeability via cell membrane. It may cause substantial toxicity, primarily due to the increased surface area of NPs. The cytotoxicity of NPs changes considerably with their size and nature of component from which they are constituted. It was described that small GNPs with minor changes in size (1.2, 1.4 and 1.8 nm) may possibly bring considerably altered cell responses [28]. The GNPs of 1.2 nm size were found to be extremely toxic and NPs more than 15 nm were relatively non‐toxic. It was also found that TiO_2_ and carbon black NPs caused inflammation and destruction to epithelial cells in rat lungs to a larger amount in comparison to their larger ones. With Zinc oxide NPs, it was detected that smaller NPs cause more toxic effect than larger ones in A549 human lung epithelial cells. In a study by Jin et al., it was found that the smaller sized ZnO NMs exhibited slightly more toxicity in comparison to large sized NMs in human neuroblastoma cells (SH‐SY5Y) [29].

## 3 THE EFFECT OF COATED AND UNCOATED NPS ON NANO–BIO SURFACE INTERACTIONS

**FIGURE 2:**
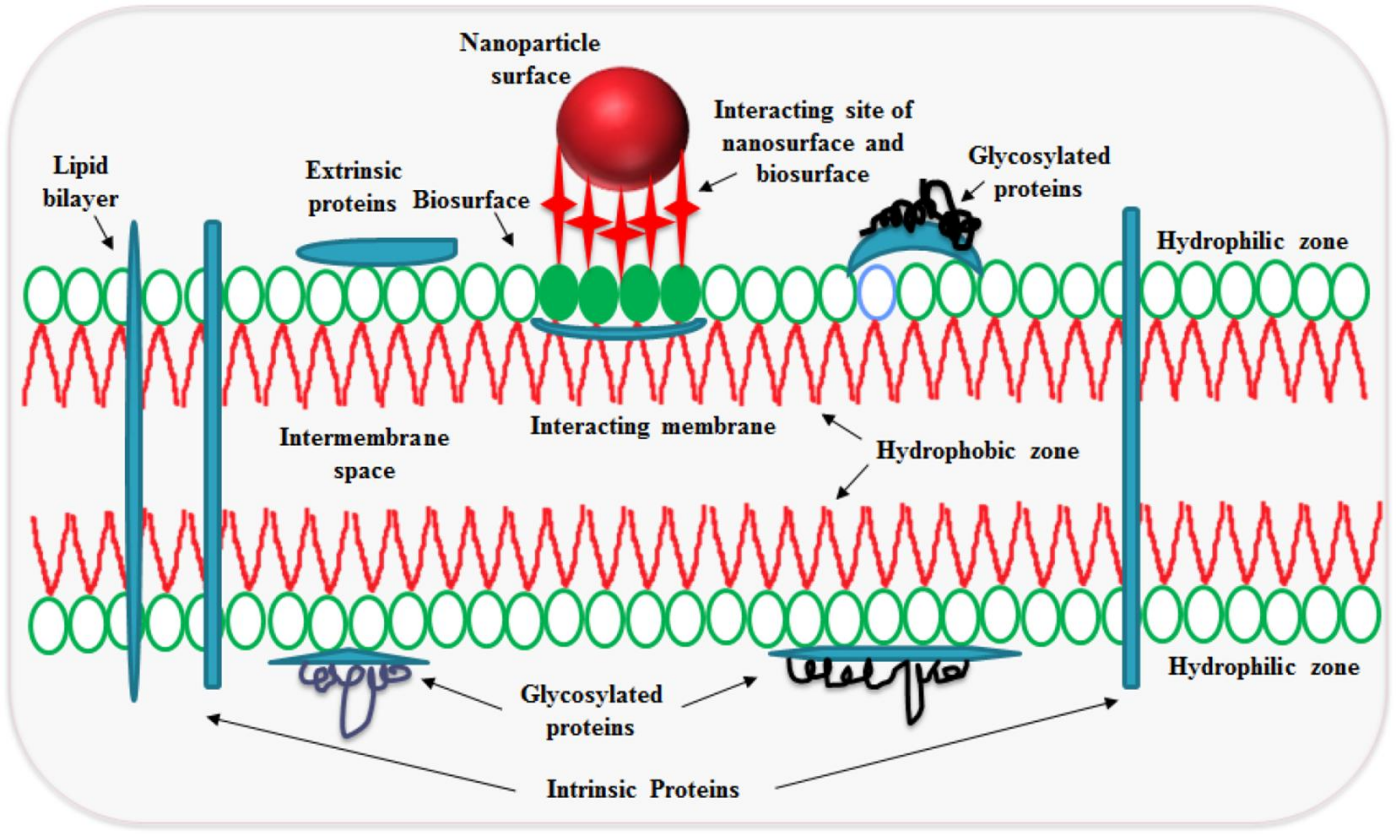
Diagrammatic representation of nano–bio surface interaction of NMs: The modified surfaces of solid NPs and hydrodynamic nature of membrane, forces and membrane bound molecules influence the interaction that activates between the nanosurface and the biosurface before internalisation

Most of the biological progressions occur at the nano–bio surface; therefore, the designing and presence of molecules on the natural biosurface is necessary for the proper interactions. Both the nanosurface and biosurface are interactive and essential in generating the biomimetic NPs for efficient delivery (Figure 2). The NPs are better suited for crossing the cell membrane by several routes and their properties play important part in relation to their entry in the mammalian cell membrane. The hydrophilic ‘head’ group and hydrophobic ‘tail’ group of the membrane have a hydrophobic layer (2–3 nm) in the middle of the bilayer acting as an obstruction for hydrophilic molecules while the outer surface resists the entry of hydrophobic molecules. The hydrophobic core mostly interacts with hydrophobic molecules/drugs, that is, for the encapsulation, while the outer hydrophilic surface is used for loading the information. Nano–bio interface involves specific and non‐specific interactions of the ligand surface with the receptor surface, wrapping of membrane surface moieties at the time of crossing of biological barrier. Inside the biological fluids, proactive, active and resistive forces operate and then NPs interact with structural and functional components, lipids molecules, proteins, enzymes, DNA etc. inside the cell.

Instead of disrupting the membrane, there is an alternative to engineer the NPs’ surface, which interacts specifically with the targeted cell. Fluorescently labelled NPs with *α*v*β*3‐integrin targeting on lipid‐coated liquid perfluorocarbon emulsions were utilised to deliver lipophilic substances to target cells without the need for entire NP internalisation [30]. Dyawanapelly et al. mentioned that the coated NPs were found to be less toxic, have greater dispersibility and demonstrate better conjugation/interaction with other bioactive molecules compared to bare NPs [31]. The studies have been carried out to study the interactions, drug delivery in the lipid membrane and to transfer the drug to the cytosol using lipid raft channel. Lipid‐coated liquid perfluorocarbon emulsions were developed to deliver the drug to the membrane without the internalisation of the whole of the NPs due to the interaction of *α*_v_ *β*_3_‐integrin expressing C32 melanoma cells [32]. A thermoresponsive polymer poly(N‐isopropylacrylamide) polymer was reported by Lombardo et al. to form a strong conjugate with the dimyristoyl phosphatidylcholine vesicles, while its NPs were found less interactive and cross the membrane without forming the conjugates [33].

Metallic and polymeric NPs have unique physicochemical properties like small size, large surface area, photo thermal outcome etc. [22]. The surface of polymeric NPs is quite different and less sensitive due to the use of biocompatible and biodegradable polymers. The polymeric spherical NPs show distinct affinity for cellular materials as compared to metallic spherical NPs [23]. In case of one‐dimensional nanostructure (nanowires and nanotubes), a high local stress and penetrating force is generated due to small contact diameter (∼1–100 nm) on membrane surface. The punctured plasma membrane using nano‐needles and nanotubes decorated with quantum dots allowed the cells to grow and divide normally [34]. The functionalised surface of titanium oxide (TiO_2_) NPs lead to destruction of cancer cell membrane and ultimately cell death. TiO_2_ NPs functionalised with –NH_2_ and –OH groups shown to have higher toxicity than –COOH group. The –NH_2_ and –OH capping altered the membrane charge, integrity and functions, while –COOH groups protected the nanosurface from unwanted interactions during the internalisation of NPs [35]. Doped‐TiO_2_‐NPs were found to induce cytotoxicity and cell cycle arrest in various tumour cells. Human lung carcinoma (A549) and breast cancer cells (MCF‐7) treated with TiO2‐ and Ag‐TiO_2_ NPs exhibited reduced cell viability, thereby indicating the chemotherapeutic effects of these NPs. It has been reported that Ag‐coated TiO_2_ NPs induced high leakage of lactate dehydrogenase in HepG2 cells causing damage to the cell membrane, whereas pure TiO_2_ NPs did not show such induction. In a study, 30‐nm‐sized GNPs bound with tumour necrosis factor resulted in the enhancement in the presence of GNP in liver and spleen, and maintained it even after 1 month [36]. The covering of the citrate ligand on the iron oxide NPs exhibited maximum interaction with the cell membrane.

The capping materials on the surface of NPs rendering them toxic or non‐toxic depend upon different types of interactions occurring within the nanosurface and biosurface. The coating of lauric acid on magnetic NPs lessened the toxicity and enhanced the uptake of NPs in comparison to the dextran‐coated magnetite NPs in mouse fibroblast and human cervical carcinoma cancer cell lines [37]. Hence, it is evident that an imperative role is being played by the coating in modulating the biocompatibility and cellular interaction of magnetic NPs. The surface coating of albumin (blood protein) at superparamagnetic NPs lessened interaction with membrane and enhanced the uptake inside the macrophages in comparison to HeLa cells [38]. The coating of superparamagnetic iron oxide NPs’ surface with synthetic polyvinyl alcohol (PVA), carboxylate‐functionalised PVA, thiol‐functionalised PVA and amino functionalisation have shown different levels of toxicity for melanoma cells [34].

The freely delivered NPs produce toxic outcome due to their interaction with plasma membrane while manual puncturing avoid the interactions. The physicochemical factors and the presence of large organic molecules (protein and DNA etc.) on the surface of NPs tend to alter the nano–bio interaction and directly or indirectly kill the cancer cells. The GNPs interact with vascular endothelial growth factor (VEGF165) and disrupt the function of proteins [22]. This gave the idea of targeting the diseased cells influenced by proteins. Transferrin‐covered round GNPs (14  and 50 nm size) indicated higher uptake rates than transferrin‐covered bar like gold NPs.

## 4 THE CELLULAR UPTAKE AND INTERACTIONS OF NPS WITH PROTEINS

Inside the cellular system, different vital processes (DNA replication, protein synthesis, signalling and endocytosis) occur for the proper functioning of cell. Recently, formation of new NMs and their biological interactions with cells, membranes, proteins, DNA, vesicles, biological fluids and other organelles have found applications in various scientific areas like health, medicine, physics, chemistry, and tissue engineering [31]. The entry of NPs inside the biological fluid opens up new interactions leading to the adsorption of proteins from the physiological environment on the surface of the NPs that form corona thus changing the properties on the NPs’ surface and also their future in the cell system. NPs in turn alter the properties of adsorbed proteins and can cause inactivation of proteins [39]. Gupta et al. explained that hard corona is formed by irreversible adsorption of proteins on NPs and that formed by reversible adsorption of proteins is called soft corona [40]. As protein corona is formed, there is a change in the physicochemical properties and biological distinctiveness of NPs. Thus, to find out the probable different impacts of physicochemical and thermodynamic interactions of NPs, the NPs and protein interactions should be properly characterised which is very much challenging.

It is also important to understand the mechanism of formation of corona to recognise the role played by corona for the interaction with NPs and it will be helpful for finding in vitro nanotoxicity and nano–bio interactions. As determined by Franqui et al., the hydrophobic interaction of the single layered graphene oxide (SLGO) and the multi‐layered graphene oxide (MLGO) showed quite different properties in a cell culture medium and detected 115 and 11 proteins, respectively, on their hard corona composition. It was found that foetal bovine serum proteins with SLGO play important roles in metabolic processes and signal transduction, however proteins enriched with MLGO found to play role in cellular development, lipid transport and metabolic processes [41]. This is due to the differences in their surface chemistry, aggregation properties and the surface area of NMs.

Owing to the continuous adsorption and desorption of proteins, the nano–bio interface shown to have alteration with time on contact with blood plasma [3]. The arrangement of adsorption of blood proteins to NPs is active, and the abundantly found proteins like albumin and fibrinogen could primarily get attached to the surface and afterwards may be substituted by other proteins which are having more affinity for the surface. The pattern of plasma proteins getting attached to single walled carbon nanotubes (SWCNT) was fibrinogen, then immunoglobulin, transferrin and then albumin. The process by which the protein molecules organise themselves on the NP surface might have impact on the biological activity of NP at the cellular level as well. Plasma proteins like human serum albumin and transferrin are displayed in order to get adsorbed as a single layer form on the iron platinum NPs’ surface. A monolayer was formed by BSA molecules were adsorbed on aluminium oxide surface by using 30%–36% of its total negative charge and adsorption of more BSA molecules were stuck to this monolayer as dimers. It was earlier shown that corona formation could interfere with the targeting of transferrin conjugates of NPs; binding to both transferrin receptors on cells and was significantly reduced in free solution [42]. The fibrinogen protein on acrylic‐acid‐coated GNPs activated the MAC‐1 receptor pathway on macrophage‐like cells and subsequently caused the inflammation response [43]. Liu et al. found that the transcytosis of albumin bound paclitaxel occurred when the glycoprotein (gp60) interacted with NPs and gave the indication of their internalisation [44].

Most of the NPs have been targeted by the cellular system for their elimination and it is an important concern for the design of NPs. This is understood that the creating polymer coating on NMs makes them highly stable and hydrophilic owing to the occurrence of large number of varied functional moieties on the NPs’ surface that play an important role in binding with the proteins [31]. The surface of TiO_2_ nanorods and nanotubes differentially adsorb the plasma proteins. There are some more challenges after designing of NPs, like binding of proteins and formation of corona on the nanosurface that activate the opsonisation, phagocytosis and the complement pathways.

The formation of corona is complex due to the stability and the binding nature of proteins [8]. The protein conformation on the surface of NPs depends upon the nature of proteins, surface of NPs and on their characteristics. The NPs’ properties such as materials shape, size, charge, and hydrophobicity influence the protein binding [45]. The major plasma proteins that is human serum albumin, immunoglobulins, and fibrinogen bound to the surface of polymeric NPs, liposomes, iron oxide NPs and carbon nanotubes.

The TiO_2_ NPs may be well absorbed by proteins in the cytoplasm in tumours that could be the reason for proteogenomic disruption. The intensities of SOD1, SOD2, heame oxygenase‐1 and *β*‐actin proteins were considerably lessened in human breast cancer MCF‐7 cells that were injected with pristine or Zn‐TiO_2_ NPs [41]. The interaction of TiO_2_ NPs with VE‐cadherin (vital for upkeep of cell shape and stability) might bring mechanical tension that results into actin reorganization and alterations in the form of plasma membrane or homophilic damage [46].

A sufficient thermodynamic energy on NPs allow the wrapping of different proteins in relation to their charge and their uptake by macrophages. The charged NPs adsorb proteins on the basis of their isoelectric point [47]. The protein binding can be altered by changing the surface and charges of NPs. The electrostatic binding of lysozyme with low molecular weight chitosan coated iron oxide NPs was favoured at pH 9.0 [48]. The unequal half‐lives of different charged NPs are due to the interaction with serum proteins such as immunoglobulin, lipoproteins, acute phase proteins, metal‐binding, sugar binding and complement proteins and coagulation factors. The binding of proteins on the surface of NPs and the interaction of adsorbed proteins with other cellular components are still not completely understood that display the differences in the biodistribution, biocompatibility, uptake and therapeutic efficacy of NPs. At the NPs surface, the soluble proteins lose their native folds and convert into the large insoluble amyloid fibrils producing toxic effects and causing amyloid diseases [22].

As amino acids are the building blocks of the proteins, the interactions between the amino acids and carbon nanotubes is important to understand the complex systems. The relation between the single‐wall carbon nanotubes (SWCNTs) and human serum proteins found an aggressive binding of these proteins with varied adsorption limits and stuffing modes. The π‐π stacking and H‐π associations amongst SWCNTs and aromatic amino acids (tryptophan, phenylalanine, and tyrosine) found to play a part in deciding the adsorption limits [49]. The binding of proteins on the NPs surface modify the properties and interactions of NPs. The interaction of proteins and NPs for the therapeutic applications depends upon the type and amount of proteins that is important for biocompatibility and uptake of NPs towards normal and cancer cells. The interaction of C3‐protein and IgG protein with lecithin coated polystyrene nanospheres helped in increasing the hepatic uptake by Kupffer cells [3, 50]. However, the assembling of complement proteins on the NPs remained unclear [45].

## 5 THE COMMUNICATION OF TARGETED NPS WITH CANCER CELLS

**FIGURE 3:**
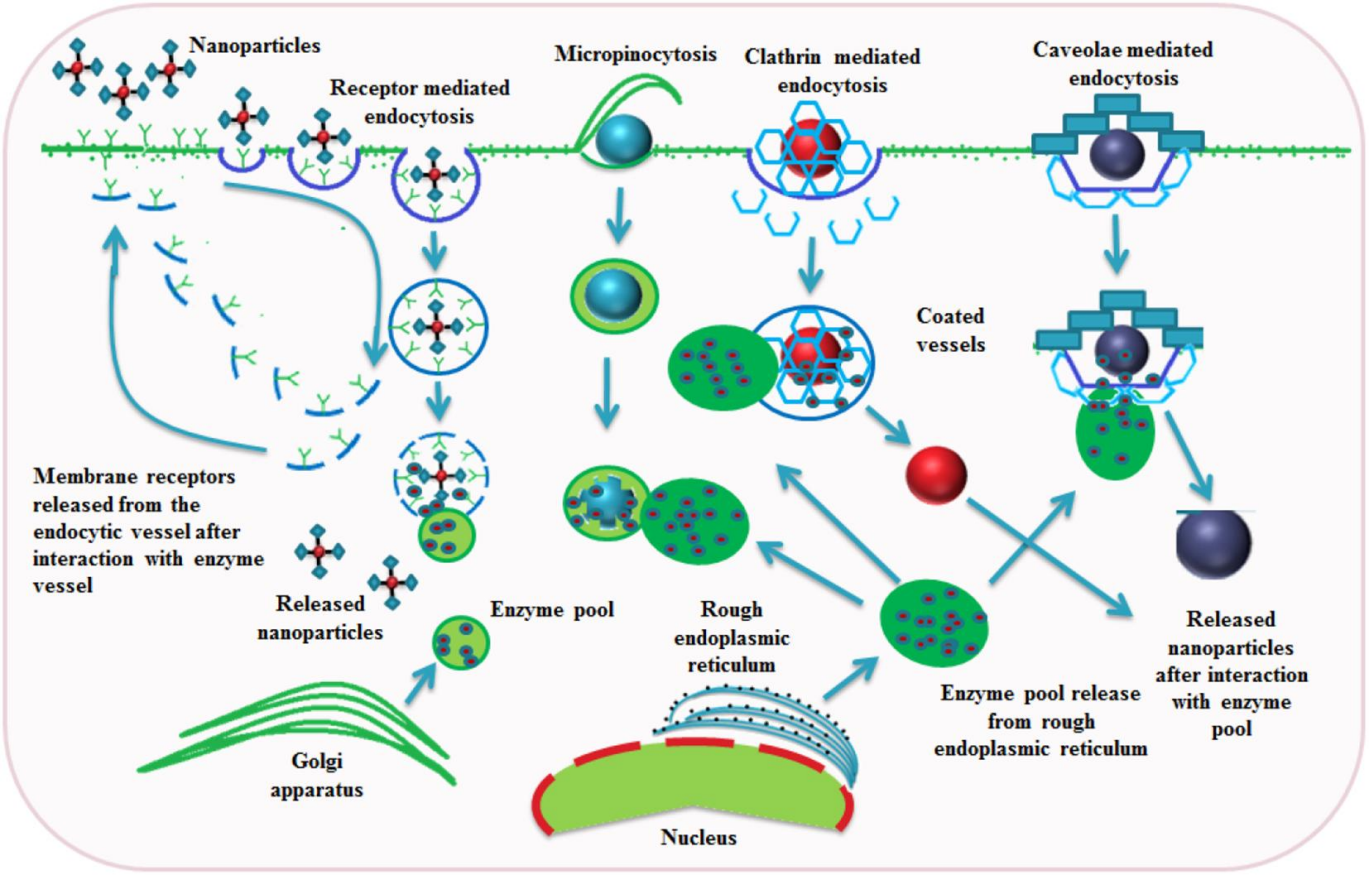
Several routes of internalisation are followed by the NPs: The receptor mediated interaction is activated by the interaction between ligands and receptors. The internalisation starts on the membrane's surface, and the cargo releases the NPs and membrane receptors after contact with the digestive enzyme. The other types of endocytosis are monitored by the specialised proteins that formed special vessels on the surface of the membrane

The cancer cells have their own molecular interactions which expressing more receptors (p‐glycoprotein, enhance folate receptor and EGFR), biomarkers (*α*‐methylacyl coenzyme A racemase) and proteins (hepsin, Pim‐1, protease/KLK4). The tumourgenesis enhances the surface charge, alters the cells fluidity, and changes the microclimate of cancer cells [51]. The tumour’s leaky nature and NPs’ enhanced permeability effect open up the passive targeting. According to the physiochemical characteristics of the nanocarrier and the nature of the targeting, the main internalisation pathways for NPs are phagocytosis, endocytic pathways like clathrin and calveolae‐mediated endocytosis, clathrin and caveolae‐independent endocytosis and macropinocytosis (Figure 3), these were very well discussed by Behzadi et al. [13]. Clathrin‐mediated endocytosis is the most studied mechanism of receptor‐mediated internalisation and probably the most widely used by almost all cell types for the internalisation of macromolecular and nano‐sized materials [52]. Caveolae‐mediated endocytosis is a highly regulated process involving complex signalling, driven by the cargo itself. After binding to the cell surface, particles move along the plasma membrane to caveolae invaginations, where they are maintained through receptor‐ligand interaction. Macropinosomes endocytic pathway does not seem to display any selectivity, but is involved in the uptake of drug nanocarriers and is expected to be responsible for the uptake of PEGylated poly‐lysine‐compacted DNA NPs [53]. The positively and negatively charged NPs use several pathways to get internalized and translocated across the colon carcinoma Caco‐2 monolayers. The positively charged NPs showed greater toxicity and cell uptake in comparison to the negatively charged NPs. The positively charged NPs use clathrin pathway for cellular internalisation and transport, whereas the negatively charged NPs utilise lipid raft pathway. Macropinocytosis plays a part in the uptake of both positively and negatively charged NPs [8].

Possibly the best known receptors adopting these pathways are transferrin, the low‐density lipoprotein receptor, the epidermal growth factor receptor and the G‐protein coupled receptors *β*_2_‐adreno receptors [26]. For receptor‐mediated endocytosis, the physicochemical properties of the ligands coating play very important roles [13]. These properties may lead to weak attachment, partial engulfment, or total engulfment of NPs. The engulfment degree of NPs is not due to the increasing strength of the receptor‐ligand interaction but changing the ligand density or hydrophobicity/lipophobicity of ligands alter the engulfment results. The nature and density of the ligands on NPs’ surface can affect the circulating half‐life from few minutes to several hours, their biodistribution as well as faster uptake. The functionalised NPs escape more rapidly and more efficiently from late endosomes and result in 10‐fold higher intracellular delivery [54]. Smith et al. developed an approach for in situ programming of leukaemia‐specific T cells using synthetic DNA nanocarriers [55].

Musallia et al. reported that the large sized chitosan NPs with greater folic acid contents and zeta potentials showed more cellular internalisation in human melanoma cells. When these NPs were decorated with high folic acid contents, it hampered NPs interaction with the cell and resulted into lesser cellular uptake of NPs. Oligochitosan NPs when decorated with folate (20% w/w) complemented the cellular internalisation. Coating of oligochitosan NPs with carboxymethyl‐5‐fluorouracil and folate (20% w/w) helped NPs binding with folate receptor, cellular internalisation and also cancer cell death [56]. The coating of the PEG layer is safe and produces the immunogenic response inside the cellular system [57]. PEGylation reduced the uptake of anticancer liposome (Doxils) by phagocytes; there was an increase in the half‐life of liposomes loaded drug. On the other hand, the NPs undergo more uptake by phagocytes when these are charged or have a hydrophobic surface thus attracting complement proteins towards themselves. The ligand 50% PEG‐NH2/50% glucose on the surface of GNPs showed 18 times rapid uptake in comparison to the NPs carrying only PEG‐NH2 or glucose in colorectal cancer cells in vitro [58]. The NPs’ surface coated with didodecyldimethylammonium bromide showed higher interaction with cancer cell lipids that was 6.7 times higher than with unmodified NPs and 5.5 times higher than with endothelial cell membrane lipids [59].

**FIGURE 4:**
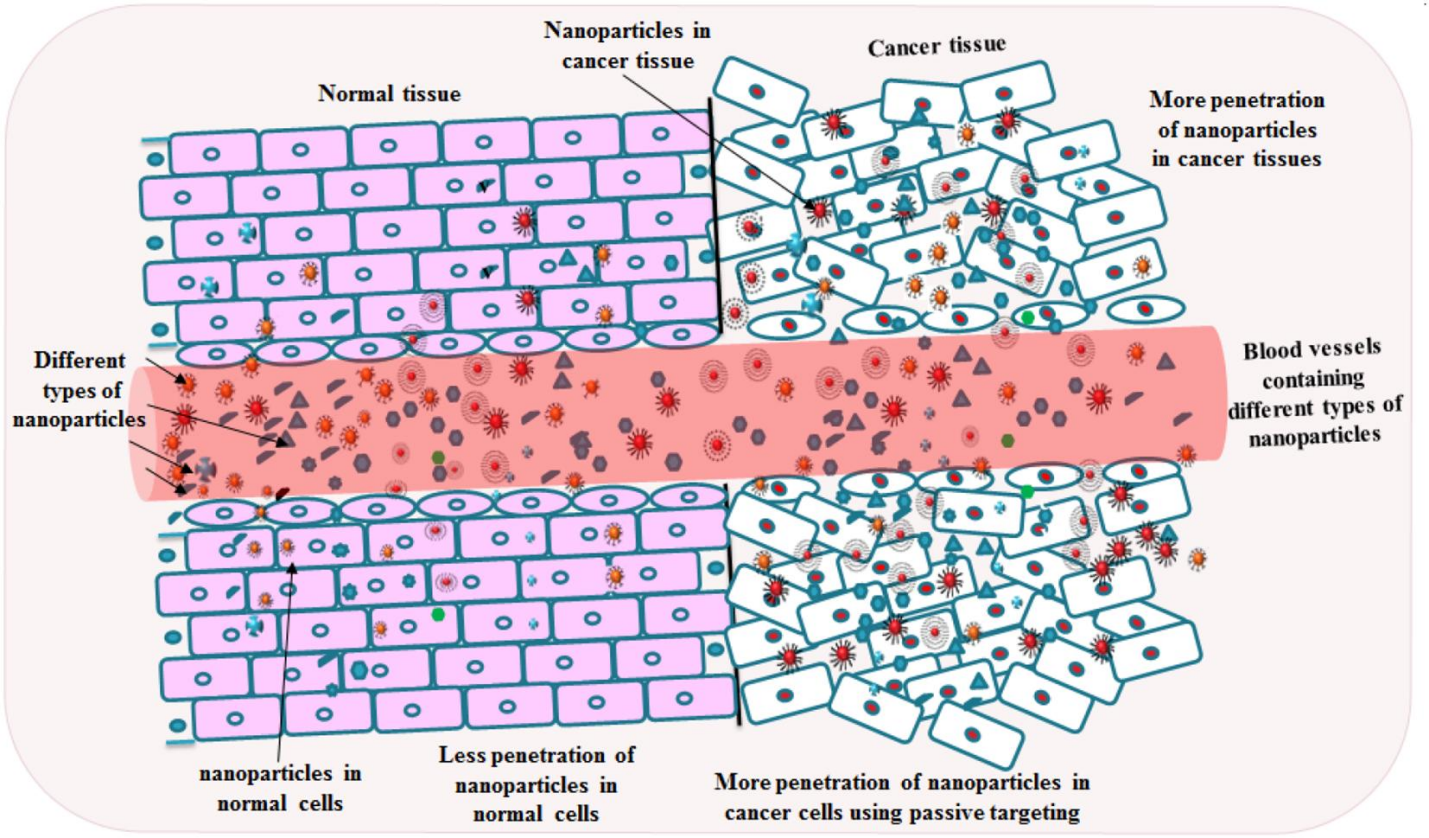
Communication of several NPs with normal and cancer tissues: mostly NPs enter in cancer tissue but presence in normal tissue concern about the unspecific interaction and internalisation. However, due to enhanced permeability retention effect, a passive targeting is operated which improves the NMs' concentration at the cancer site, but not constantly. However, the perfect targeting does not reach the cancer site several times, due to energetic resistance generated by the natural system

The Ca^2+^ inflow into the extracellular atmosphere can be increased on continuous exposure to pristine and modified TiO2 NPs that occur via membrane L‐type Ca^2+^ channels and increased expression of the PKC/p‐38 MAPK cascade, eventually stimulating NF‐κB. TiO2 NPs might break cancer cell integrins that start cell death by disturbing the metabolic routes [46]. The enhanced cellular internalisation interaction rather than an increased tumour accumulation is responsible for the antitumoural efficacy of actively targeted nanocarriers [55]. The presence of multiple copies, uniformity and longer ligands of tumour‐specific or antibodies would increase the interaction for binding and enhancing the tumour‐targeting specificity, rendering minimal drug toxicity in normal tissues [60]. The particle can be totally engulfed if the hydrophobic/lipophobic properties of ligands are properly designed. Even in the absence of targeting ligands, drug delivery systems can be engineered to target a particular cell, or non‐specifically absorbed by diseased tissues by optimising their biophysicochemical properties. However, when particles extravagate out of the vasculature into the tumour tissue, their retention and active targeting and receptor‐mediated endocytosis facilitate specific uptake by cancer cells (Figure 4). This process could result in higher intracellular drug concentration and increased cellular cytotoxicity. Few common targeted receptors express on healthy tissue and result in nonspecific targeting and subsequently increase the toxicity [61]. An antibody that only binds overexpressed, mutants, or ligand‐activated forms of EGFR in cancer cells was identified by screening thousands of EGFR monoclonal antibodies for tumour specificity. Phase I clinical trials using a humanised A33 monoclonal antibody (huA33 mAb) shown promising results in targeting colorectal tumours, and cancer cells showed slower A33 turnover rates in comparison to the normal intestinal epithelial cells [5]. The anti‐EGFR antibody conjugated on colloidal GNP specifically and homogeneously binds to the surface of the cancer cells with 600% greater affinity than with the non‐cancerous cells as evident by Surface Plasmon Resonance Imaging and Spectroscopy in two malignant oral epithelial cell lines [27]. The reports are available with a relatively modest improvement in tumour tissue accumulation of targeted drug delivery systems relative to non‐targeted drug delivery [62]. EGFR‐targeted Fe_3_O_4_@TiO_2_ NPs interacted with karyopherin‐*β*, a protein that is critical for the translocation of ligand‐bound EGFR NPs to the nucleus, as observed by flow cytometry [63]. Eivazi et al. studied that the conjugation of doxorubicin loaded PLGA NPs with anti‐EGFRvIII antibody showed more selective uptake of NPs into U87 MG vIII cells than non‐conjugated NPs [64]. The cytotoxicity of the antibody conjugated doxorubicin loaded PLGA NPs was found to be more than BSA‐DXR‐PLGA NPs against U87 MG vIII cells confirmed by apoptosis, MTT and cell cycle assays.

Heinz et al. reported that the cellular uptake of trastuzumab‐protein‐coated polystyrene NPs were utilised in breast cancer cells [57]. Hybrid poly (ethylene imine) mesoporous silica NPs were internalized five times more by HeLa cells as compared to the normal cells determined by flow cytometry [65]. Generally, the inward budding vesicles comprise receptor proteins which recognise the precise chemical groups on the molecules to be internalized. In fact, every targeting NM commonly displays no specific rule for the interactions. The proteins adsorbed to NPs trigger the cell surface receptors and readily activate the cell’s uptake machinery, whereas the adsorbed proteins that only weakly interact with membrane‐associated biomolecules will reduce the uptake of the ‘disguised’ NPs. Different studies determined the increase in in vivo drug delivery efficiency and tumour inhibition by targeting numerous receptors [63]. A study reported the missing of target (transferrin receptors) due to the interaction of active nanosurface with lots of plasma materials in the cancer cells during drug delivery [66].

Presently, NPs show their growing presence in numerous technological projects and advancements. They have various disadvantages, for example, the toxicological effects in biological systems, which may restrict their utilisation inside the medical fields. The main disadvantage of the NM is the symmetry of NM; the slight change in size (5–10 nm) or shape or charge changes the interaction of NPs. The different NMs of same size or shape or charge have different interactions and showed different toxicity with cancer and normal cells. There are reports of macrophage dysfunction, inflammatory responses and genotoxicity, hepatotoxicity, nephrotoxicity, immunotoxicity, cardiotoxicity due to NMs by Roy et al. and Madannejada et al. [28, 67]. Caixeta et al. explained that numerous NMs were specified to control parasites and can act as intermediate hosts to infections and several ignored diseases induced by many fungi, viruses, an d bacteria [68]. They have a number of disadvantages, such as the cytotoxic effects in living organisms, which may limit their use within the clinical setting.

Some important components must be considered during the nano–bio interface development such as the communication of NPs with their environment, fundamentally with different NMs and biomolecules. A report described by Auría‐Soro et al. showed antibacterial potential of Alternaria sp. synthesised silver (Ag) NPs as antibacterial material because of their high toxicity against human pathogenic microorganisms [69]. Zhao et al. also synthesised indole derivatives (tryptophan and 5‐aminoindole) and functionalised GNPs that showed antibacterial potential against Gram‐negative bacteria [70].

## 6 THE E‐DATA PORTALS OF NANOMATERIALS

The growing significance of NMs in different fields has brought an upsurge in their synthesis and development. Green synthesised NPs have been used for the removal of pollutants either by their reduction (of p‐nitrophenol and congo red dye by Au/Ag or Ag NPs) or by degradation (of reactive Green 19 Dye by TiO_2_ NPs) [71, 72]. Currently, a lot of NMs are synthesised and enormous amounts enter into our ecosystems, having significant impacts on biodiversity. Moreover, due to their immediate impacts on the climate, NMs are transported between various organisms through the food web and have shown secondary effects to living beings at higher trophic levels. Therefore, accumulation and magnification of NMs in biosystems may cause substantial threats to the climate and human health.

A wide range of data is currently generated on chemical, physical and biological properties of newly synthesised NPs based NMs. However, there is dearth of research data that is available from electronic portals or generated from research laboratories on these NMs [73]. Thus, various e‐data portals and practices are being formed to collect, manage and share the data and knowledge through open resources. The generation of these open resources will help in synthesis of new nanomaterials rationally using nanoinformatics tools with reliable knowledge. Many e‐data portals tools manage and share the knowledge of NMs through open resources. A few important data portals already working in this area, such as Nanomaterial‐Biological Interactions knowledgebase, Oregon Nanoscience and Microtechnologies Institute, Cancer Nanotechnology Laboratory, Nanomaterial Registry, ISA‐TAB‐Nano, OECD Safety of Manufactured NPs and Nano‐EHS database Analysis Tool, are described in this section .

### 6.1 Nanomaterial‐Biological Interactions Knowledgebase

Nanomaterial‐Biological Interactions (NBI) knowledgebase, established by the Environmental Health Sciences Centre and Oregon Nanoscience and Microtechnologies Institute is a freely accessible database useful in annotation, characterisation, functionalisation, agglomeration state, synthesis techniques of NMs and NM‐natural connections at different levels of natural associations (e.g. atomic, cell or organism) [74]. NBI intends to alert different offices about objectives to recognise the practical spaces and outline standards for high performance, natural favourable NMs and biological effects of on exposure NMs. NBI knowledgebase permits unbiased explanations of NM and biological interactions, distinctive structural features that direct interactions of nanomaterial and biological surface, to find the procedures that can project NM‐biological interactions, and data collection to predict the after effects of nanomaterial exposure [75].

### 6.2 Oregon Nanoscience and Microtechnologies Institute

Oregon Nanoscience and Microtechnologies Institute (ONAMI) presents the customary and regular enterprises, where developed research and commercialisation can enhance personal satisfaction without natural hazards [74]. It involves Oregon’s three research universities―Oregon State University, Portland State University and University of Oregon, research institutes and researchers. It is the Research Centre aiming for the growth of research and commercialises it to speed up the innovation and technology related commercial progress in Oregon and the Pacific Northwest. Centre of ONAMI is to create guidelines utilising NPs of Green science and to bolster the NBI knowledgebase.

### 6.3 Cancer Nanotechnology Laboratory

Cancer Nanotechnology Laboratory (caNanoLab) started in 2007 is an information sharing entryway [73] and online data repository which intends to provide platform to analyse and share the data among researchers working in the area of cancer using nanotechnology techniques [42]. caNanoLab was planned as a mean to be useful with other resources of nanotechnology techniques used. caNanoLab permits research analysts to get facts on characterised NMs that include composition, experimental characterisation, allied researches and for representation of NMs annotation, allots work in the light of in vitro and in vivo methodologies and offer nanotechnology protocols. Researchers can also download reports of composition, physical and pharmacokinetics properties, characterisation, toxicity, and functions of NMs. caNanoLab was formed for reports and data pool to affirm the formation of comprehensively identified nanomaterials, the description of standard protocols, the collection of records and documents from varied description reports and results.

### 6.4 Nanomaterial Registry

As there is a shortage of e‐data portals which provide information about the NMs, Nanomaterial Registry found in 2010 is the information driven entryway that enable the nanotechnologists and analysts to limit this gap in an advantageous and intact way [73]. This is a widely accessible resource which is formed through associations of various participations by nanotechnology groups, involving, governing bodies, industries, and the academic world. This was established as a valuable mean to develop naturally constructed on a cyclic association with the nanotechnology clusters. This information base is useful in observing the various properties of NMs and allows storing it on our desktop. The Nanomaterial Registry Portal at nanoHUB.org intends to contain all nanomaterial natural/ecological investigation information and enables the clients to draw data records in view of NMs assay and quality [76]. In the future, this will show a tremendous potential for important long‐lasting effects that involve helping in the progress of protocols, tests, techniques, measures, and constructing procedures; fastening up the transformation of novel NPs and NMs for biomedicine‐ and environmental‐related purposes; encouraging measures in screening, examining, analysis, management, and disposal of NMs.

### 6.5 ISA‐TAB‐Nano

ISA‐TAB‐Nano document designs give a general and adaptable structure to record and incorporate NM representations, convention data and test information (e.g. metadata and endpoint approximations) [77]. The ISA‐TAB‐Nano files containing the information are in the form of four file formats‐investigation file, study file, assay file and material file to represent the information connected to the description of nanomaterials identity, their synthesis, characteristics, applications. The data is created on the premise of four spreadsheet‐based document configurations or TAB‐delimited records obtained from the NPs [78]. Three file formats (Investigation, Study and Assay files) are adjusted from the well‐known ISA‐TAB description; while the material file format is developed in de novo to describe the complication of NPs and associated small molecules more readily. To receive people's response, ISA‐TAB‐Nano particularly has been submitted as ASTM (American Society for Testing and Materials) direction of work, which provide data for the progress of society. Mostly the data obtained from various non‐standard sources and reports are generally insuffieicnt and produce unorganized data sharing and lack experimental reproducibility. Therefore, there is difficulty in understanding, interpretation and evaluation of nanomedicines utilising such scattered informations.

### 6.6 OECD for the Safety of Manufactured NMs

OECD Test Guidelines for the Safety Testing of Chemicals propose for proper evaluation of NMs and sometimes few rules ought to be adjusted for the particular properties of NMs. The OECD Working Party on Manufactured Nanomaterials offers a worldwide medium to talk about problems and concerns regarding nano‐safety. They have searched the requirement to accommodate and adapt the existing OECD Test Guidelines and Guidance Documents in addition to producing new guidelines and documents to specially deal with nanomaterial problems [79]. Mutual Acceptance of Data (MAD) in the evaluation of chemicals proceeds consistently with €150 million OECD spares. The countries in the MAD framework are Argentina, Brazil, India, Malaysia, Singapore, South Africa, with Thailand being a temporary follower. The arrival of MAD to NPs will fundamentally lower the potential for non‐levy work obstructions between nations while advertising manufactured NPs or items that incorporate NPs and share the workload between nations in testing and surveying all the NPs which are available in the market. We know that the NPs of zinc and silica have been used in sunscreens and beauty care products, and so their hazards should be precisely evaluated. The OECD has been working in nanoscience for hazard evaluation of the produced NPs, which indicate new characters at the nanoscale [79].

### 6.7 Nano Environment, Health and Safety Database Analysis Tool

Nano Environment, Health and Safety (EHS) database is a major work for human and environmental safety as the nano companies/organisations are developing engineered NMs which can absolutely affecting people and the environment. Thus, these companies/organisations have main accountabilities related to the development of the engineered NMs [80]. This databsae allows two ways analysis of data base literature in terms of the International Council on Nanotechnology’s (ICON) Environment, Health and Safety (EHS) database [81]. In two ways on the ICON web site, the first is a Simple Distribution Analysis (pie chart) and second type is a Time Progressive Distribution Analysis (histogram). This asset additionally enables us to produce and export custom reports, and in addition having the capacity to tap on the response to create a rundown list of publications that meet our inquiry criteria. ICON constantly updates the EHS database. EHS will emphasize on the kinds of NMs that are possibly present in the environment, and bring about the biological and chemical conversion of engineered NMs for environment safety [82].

## 7 CONCLUSION

The NPs are used for drug delivery through the guidance of different antibodies/ligands. The nanosurface is quite active and its interaction is controlled by different forces appearing on both the nanosurface and biosurface leading to the change in protein structure and membrane properties, and may damage the cell organelles. We can regulate these forces for proper interaction of normal and cancer cell tissues by applying coating materials of different charge/size/proteins on the surface of NPs during or after synthesis. Before projecting the NPs, their surface energy should be checked and detected by various techniques and protocols during in vivo and in vitro conditions. Scientists and technologists need to understand how to regulate the interaction between nanosurface and biosurface by nanotechnological approach that could bring a change in the proactive targeting and active targeting. The interaction of NPs with the cellular system should be completely considered, checked and reported, otherwise this could result in serious toxicity trials. Therefore, the concern about their possible adverse effects and potential toxicity is required, as they are new and hardly investigated, mainly for their interactions with biological systems. As there is less availability of data on NMs, few e‐data portals working in this field are also discussed. Today, various e‐data portals are available in the form of open information, which easily explain the risk and properties associated with the synthesised NPs. The digital data approach increases the efforts of researchers for taking up the current challenges and complete them in a time bound manner. Based on nano–bio interface interactions, the developed nanostructure can be smartly filled with novel information and sophisticated topographies to treat the diseases in better dimensions.
